# Non-Invasive Multimodal Neuromonitoring in Non-Critically Ill Hospitalized Adult Patients With COVID-19: A Systematic Review and Meta-Analysis

**DOI:** 10.3389/fneur.2022.814405

**Published:** 2022-04-14

**Authors:** Denise Battaglini, Lavienraj Premraj, Samuel Huth, Jonathon Fanning, Glenn Whitman, Rakesh C. Arora, Judith Bellapart, Diego Bastos Porto, Fabio Silvio Taccone, Jacky Y. Suen, Gianluigi Li Bassi, John F. Fraser, Rafael Badenes, Sung-Min Cho, Chiara Robba, the COVID-19 Critical Care Consortium

**Affiliations:** ^1^Anesthesia and Intensive Care, San Martino Policlinico Hospital, IRCCS for Oncology and Neuroscience, Genoa, Italy; ^2^Department of Medicine, University of Barcelona, Barcelona, Spain; ^3^Griffith University School of Medicine, Gold Coast, QLD, Australia; ^4^Critical Care Research Group (CCRG), Herston, QLD, Australia; ^5^Faculty of Medicine, The University of Queensland, Herston, QLD, Australia; ^6^St. Andrew's War Memorial Hospital, Uniting Care Health, Spring Hill, QLD, Australia; ^7^School of Medicine, Johns Hopkins University, Baltimore, MD, United States; ^8^Department of Surgery, Section of Cardiac Surgery, Max Rady College of Medicine, University of Manitoba, Winnipeg, MB, Canada; ^9^Royal Brisbane and Women's Hospital, Brisbane, QLD, Australia; ^10^Department of Critical Care, Sao Camilo Cura D'ars Hospital, Fortaleza, Brazil; ^11^Intensive Care Unit, Erasmus Hospital, Free University of Brussels, Brussels, Belgium; ^12^Queensland University of Technology, Herston, QLD, Australia; ^13^Institut de Ricerca Biomedica August Pi i Sunyer (IDIBAPS), Valencia, Spain; ^14^Department of Anesthesia and Intensive Care, Hospital Clinic Universitari, INCLIVA Research Health Institute, University of Valencia, Valencia, Spain; ^15^Department of Surgical Sciences and Integrated Diagnostics (DISC), University of Genoa, Genoa, Italy

**Keywords:** neuromonitoring, hospital, COVID-19, coronavirus disease, electroencephalogram

## Abstract

**Introduction:**

Neurological complications are frequent in patients with coronavirus disease-2019 (COVID-19). The use of non-invasive neuromonitoring in subjects without primary brain injury but with potential neurological derangement is gaining attention outside the intensive care unit (ICU). This systematic review and meta-analysis investigates the use of non-invasive multimodal neuromonitoring of the brain in non-critically ill patients with COVID-19 outside the ICU and quantifies the prevalence of abnormal neuromonitoring findings in this population.

**Methods:**

A structured literature search was performed in MEDLINE/PubMed, Scopus, Cochrane, and EMBASE to investigate the use of non-invasive neuromonitoring tools, including transcranial doppler (TCD); optic nerve sheath diameter (ONSD); near-infrared spectroscopy (NIRS); pupillometry; and electroencephalography (EEG) inpatients with COVID-19 outside the ICU. The proportion of non-ICU patients with CVOID-19 and a particular neurological feature at neuromonitoring at the study time was defined as prevalence.

**Results:**

A total of 6,593 records were identified through literature searching. Twenty-one studies were finally selected, comprising 368 non-ICU patients, of whom 97 were considered for the prevalence of meta-analysis. The pooled prevalence of electroencephalographic seizures, periodic and rhythmic patterns, slow background abnormalities, and abnormal background on EEG was.17 (95% CI 0.04–0.29), 0.42 (95% CI 0.01–0.82), 0.92 (95% CI 0.83–1.01), and.95 (95% CI 0.088–1.09), respectively. No studies investigating NIRS and ONSD outside the ICU were found. The pooled prevalence for abnormal neuromonitoring findings detected using the TCD and pupillometry were incomputable due to insufficient data.

**Conclusions:**

Neuromonitoring tools are non-invasive, less expensive, safe, and bedside available tools with a great potential for both diagnosis and monitoring of patients with COVID-19 at risk of brain derangements. However, extensive literature searching reveals that they are rarely used outside critical care settings.

**Systematic Review Registration:** www.crd.york.ac.uk/prospero/display_record.php?RecordID=265617, identifier: CRD42021265617.

## Introduction

The use of non-invasive multimodal neuromonitoring to investigate the potential for neurologic derangements in patients with no primary brain injury has grown over the past decades (1). Several clinical conditions, other than primary brain injury, may have potential risk for neurological diseases, including liver and renal failure, post-cardiac arrest syndrome, severe respiratory distress, polytrauma, sepsis, and many others (1). The incidence of neurological complications in patients with coronavirus disease-2019 (COVID-19) outside the intensive care unit (ICU) varies from 2.6 to 36.4%, including mostly neuromuscular disorders, cerebrovascular events, acute encephalopathy, seizures, and miscellanea of symptoms (2, 3). Neurological complications in patients with COVID-19 occurred either as presenting symptoms or during the course of the disease (4–6). These complications frequently impacted long-term patient outcome, presenting as impaired activities of daily living, cognition deficits, anxiety, fatigue, depression, reduced return to work, and sleep-related problems (7).

Non-invasive multimodal neuromonitoring allows the detection of cerebral derangements at the patient's bedside, and its utility has been recognized both in patients with brain damage and those without primary brain injury (8, 9). The most frequently used neuromonitoring techniques to monitor the brain include transcranial doppler (TCD), optic nerve sheath diameter (ONSD), automated pupillometry, near-infrared spectroscopy (NIRS), and electroencephalography (EEG) (4–6). Despite the high prevalence of neurological manifestations and complications in non-critically ill patients with COVID-19, only a few of these techniques have been employed in this patient-cohort outside the ICU (4–6).

Therefore, the aim of this systematic review and meta-analysis was to assess the prevalence of abnormal cerebral findings detected using various neuromonitoring tools in patients with COVID-19 outside the ICU.

## Methods

### Search Strategy and Selection Criteria

We performed a systematic review and meta-analysis in accordance with the Preferred Reporting Items for Systematic Reviews and Meta-Analyses (PRISMA) guidelines (Supplementary Table 1) and the Joanna-Briggs Institute (JBI) (10) Reviewer's Manual for Systematic Reviews of Literature (11, 12). The study protocol was registered and published on PROSPERO on 5 July 2021 [(*International Prospective Register of Systematic Reviews* – PROSPERO (CRD42021265617)].

**Figure 1:**
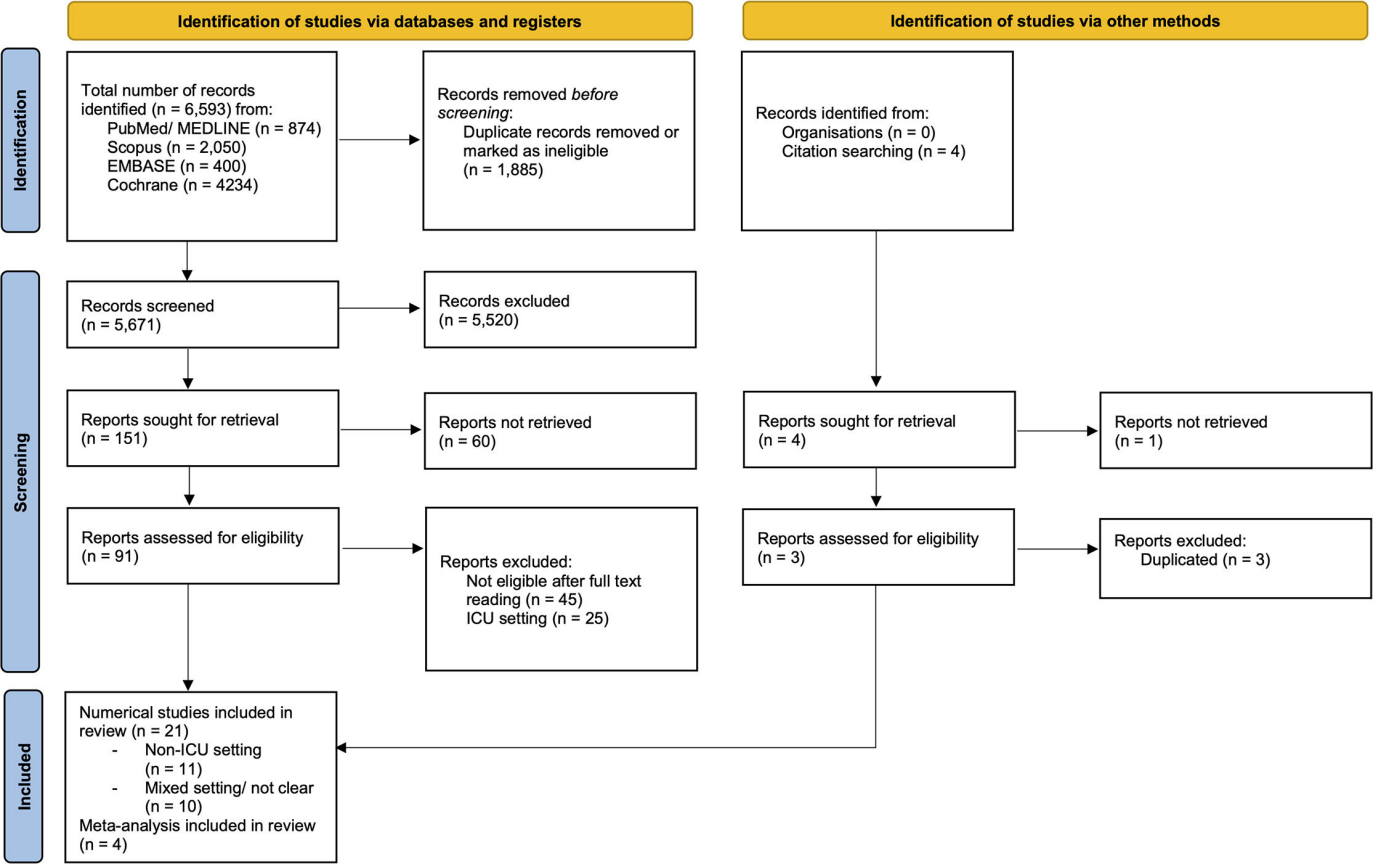
PRISMA 2020 flow diagram for new systematic reviews which include searches of databases, registers, and other sources. From (12) http://www.prisma-statement.org/.

The search was conducted on MEDLINE (National Library of Medicine: Bethesda, MD), PubMed (National Library of Medicine: Bethesda, MD), Scopus (Elsevier®), EMBASE (Elsevier®), and Cochrane (Oxford, U.K.; Vista, Calif.: Update Software Ltd.) electronic databases. Studies were also identified by citation searching from the bibliography of each relevant study, as reported in Figure 1.

All the search terms were MEdical Subject Heading [MESH] terms, including COVID-19 terms (coronavirus disease, COVID-19, coronavirus disease 2019, SARS-CoV-2, covid, coronavirus) and neuromonitoring terms (electroencephalog^*^, BIS, bispectral index, EEG monitor^*^, EEG, neuromonitor^*^, near infrared spectroscopy, NIRS, transcranial doppler, TCD, TCCS, ONSD, optic nerve sheath diameter, optic nerve, pupillometer, pupillometry” intracranial pressure, ICP, cerebral compliance, flow velocities). The search strategy used in PubMed was modified to suit other databases. The full search strategy is provided in Supplementary Material—Item 1. We included all studies that met the inclusion criteria and that had data available from 1 January 2020 to 7 March 2022. We also manually screened citations of relevant articles to identify additional studies. No language restrictions were applied.

The inclusion criteria were as follows: (1) study population of adult patients (age 18 years of age or older) diagnosed with COVID-19 by positive real-time polymerase chain reaction (RT-PCR) assay, (2) studies that described the use of non-invasive neuromonitoring in patients with COVID-19 who are not-critically ill as either a primary or secondary endpoint, and (3) observational studies, including case series with more than 10 cases, case-control studies, cohort studies, systematic reviews, meta-analysis, editorials, and letters were included for screening. The exclusion criteria were unpublished data from abstracts or congress presentations (i.e., gray literature), editorials, commentaries, letters to the editor, opinion articles, reviews, meeting abstracts, and original articles lacking an abstract and/or informative details. Studies performed in pediatric-age patients were also excluded.

The study selection started by screening titles and abstracts of articles retrieved from the search. For articles identified to be potentially relevant, the full text was then reviewed. The full text was also reviewed if a decision could not be made from reading the title and abstract alone. Two investigators (DB and LP) independently screened the titles and abstracts of retrieved articles, dividing the articles into three subgroups: “articles included” and “articles excluded” (if agreement) or “uncertain” (if disagreement). Disagreements in the study selection were resolved by consensus and further examination by two expert authors (SMC and CR). In case of multiple studies using the same dataset or cohort, we included the most comprehensive study with the largest number of participants and excluded the others.

### Data Analysis and Statistical Analysis

We used a predefined and standardized data extraction form to collect information from all the eligible studies in an electronic spreadsheet. In case of impossible extraction of the pertinent data, the corresponding authors of selected articles were contacted to obtain missing data related to trial demographics, methods, and/or outcomes. Data extraction was performed by two independent reviewers (DB and LP) using a standardized abstraction spreadsheet (Microsoft Excel, V 14.4.1; Microsoft®, Redmond, WA), in accordance with the Population, Intervention, Comparison, and Outcomes (PICO) approach.

From each eligible study, we extracted title, study design, first author, date of publication, publication type, study site, settings [non-ICU only or a mixed ICU and non-ICU population], primary outcome, the total number of subjects, the number of subjects in the ICU, demographic characteristics of subjects (age, country of residence, male/female, sample size, diagnosis, history of neurological complications, new neurological manifestations), brain images, neuromonitoring features, and risk factors for mortality or complications (Supplementary Tables 2, 3).

The quality of the studies included in the meta-analysis was evaluated by two authors (DB and CR) independently following the JBI appraisal tools criteria for defining low-, medium-, and high-quality studies. The assessment of quality varied depending on the study design and methodology. Case reports and case series were assessed for quality with the modified 8-item Newcastle Ottawa Scale (NOS) (13), while case-control and cohort studies were assessed using the COVID-19 adapted NOS (14), (Supplementary Material—Item 2).

Non-numerical data were reported in both narrative and tabular forms. Numerical data of abnormal findings on neuromonitoring were collected for prevalence analysis. Data were reported as mean (standard deviation, SD), median (1st−3rd quartile, IQR), or absolute numbers (percentage), as appropriate. Data reported as median (IQR) were converted into an estimated mean (SD) using the following formula (m = median, l = lower, u = upper, ss = sample size) (15):


Estimate mean (eMean) =   (l + 2m + u)/4 + (l-2m + u)/4ss

$$\text{Estimate SD }(\text{eSD})\;=\;1/12\{[{\left(l\;-\;2m\;+\;u\right)}^{2}/4]\;+\;{\left(u\;-\;l\right)}^{2}\}$$

The meta-analysis of prevalence was conducted for studies that reported a pooled prevalence of altered neuromonitoring features. Pooled prevalence and forest plots were obtained using dedicated software for the meta-analysis (OpenMeta Analyst®) (16). A random-effects model (DerSimonian and Laird) was used because of high levels of heterogeneity between the populations and neuromonitoring tools. Heterogeneity between studies was assessed using the I^2^, with an I^2^ of more than 75% indicating substantial heterogeneity.

### Definitions

All patients who did not require supportive critical care (i.e., invasive mechanical ventilation, hemodynamic support, organ support like renal replacement therapy or extracorporeal membrane oxygenation) and were not admitted to an ICU were defined as “non-critically ill patients with COVID-19. The proportion of the COVID-19 population suffering from neurologic derangement identified by neuromonitoring at the study time was defined as point-prevalence (17). The number of patients with COVID-19 in the selected study with a particular neurological condition identified by neuromonitoring was reported as the numerator, while the total number of non-ICU patients with COVID-19 identified in the study was reported as the denominator.

Regarding TCD derived-measures, high noninvasive intracranial pressure (nICP) was considered as >20 mmHg (18, 19). Low and high estimated cerebral perfusion pressure (eCPP) was considered as eCPP ≤45 mmHg and eCPP ≥75 mmHg (6). Abnormal pulsatility index (PI) was considered as ≥1.2 (6). Abnormal pupillary light reflex measured using the automated pupillometer was defined as a pupillary constriction rate <13% (20–22). Abnormal EEG findings were reported and classified using the terminology of the American Clinical Neurophysiology Society (23) and were categorized as (1) abnormal background abnormalities (delta/theta or alpha and beta slowing background, posterior dominant alpha rhythm, burst attenuation, burst suppression, cycling alternating pattern of encephalopathy); (2) EEG confirmed seizures (electroencephalographic seizures, electroencephalographic status epilepticus, electroclinical status epilepticus, electroclinical seizures, brief potentially ictal rhythmic discharges, ictal interictal continuum); or (3) rhythmic and/or periodic patterns (periodic discharges, rhythmic delta activity, spike and wave, or sharp and wave) (23).

## Results

### General Characteristics of Neuromonitoring in the Non-ICU Population

Spanning 6,593 articles, after eliminating duplicates and ineligible records (*n* = 1,885), irrelevant records (*n* = 5,520), reports not retrieved (*n* = 60), and reports excluded after full text reading (*n* = 45) or in ICU setting (*n* = 25), 21 studies were included for relevance (Figure 1). The selected studies were as follows: two studies on TCD in non-ICU settings (prospective observational); no studies on ONSD and NIRS outside the ICU; two studies on pupillometry (prospective observational); 17 studies on EEG findings (five from which was possible to extract data for prevalence meta-analysis including three case series and two retrospective observational studies, 12 studies from which data were impossible to extract for prevalence meta-analysis but were useful for understanding neuromonitoring features in COVID-19 outside the ICU), and four meta-analyses. The sample size varied among studies from the smallest of 10 to the largest of 197 patients.

A total of 907 (586 men and 321 women) patients were reviewed. Overall, 368 non-critically ill patients with data on non-invasive neuromonitoring (TCD, pupillometry, and EEG) were reported. Among them, 97 patients (62 males and 35 females) were considered for prevalence meta-analysis on EEG findings. Non-ICU patients' age varied between 38 and 66 years (pooled estimate mean 51.65, 95% CI 36.53–66.76); however, it was not reported in one study. Regarding the type of neuromonitoring employed in non-ICU settings, 45 patients underwent TCD, 80 underwent pupillometry, and 247 underwent EEG.

Previous neurological comorbidities were not reported in seven studies, while a high variability of presentations was found in the other studies (see Supplementary Table 2). New neurological complications were detected in several patients either by clinical signs, brain imaging, or neuromonitoring, while they were not reported in five studies. New neurological complications are reported in Supplementary Table 2. Methodological features of the included studies are reported in Supplementary Tables 2, 3. Indications for the use of non-invasive neuromonitoring tools in non-critically ill patients with COVID-19 were highly heterogenous, with the most common being neurological derangements such as altered consciousness, confusion/delirium, and clinical suspicion of seizures (Table 1). Three studies applied neuromonitoring tools solely for exploratory purposes (Supplementary Table 4).

**Table 1:** Indications for neuromonitoring and new neurological manifestations in non-critically ill patients with COVID-19.

**References**	**Indications for neuromonitoring**	**New neurologic manifestations investigated with neuromonitoring tools**
Ayub et al. (24)	Suspicion of brain involvement	Altered mental status *n* = 24, cardiac arrest *n* = 2, possible seizures *n* = 11
Bellavia et al. (25)	Exploratory reasons	**/**
Besnard et al. (26)	Suspicion of brain involvement	Confusion n=14, n=13 epileptic seizures, altered mental status n=5, delayed awakening n=6, hallucination/behavioral problems n=2, AIS n=1, meningoencephalitis n=1
Cecchetti et al. (27)	Suspicion of brain involvement	Transient loss of consciousness n=5, seizure/spasm n=5, delirium n=3, coma n=5
Corazza et al. (28)	Suspicion of brain involvement	Altered mental status n=19, seizures n=8
Galantopou et al. (29)	Suspicion of brain involvement	Altered mental status n=20, confusion n=1, gaze deviation n=2, seizure-like events n=12
Karahan et al. (30)	Exploratory reasons	**/**
Lambreq et al. (31)	Suspicion of brain involvement	Delirium n=24, seizures n=22, delayed awakening n=17
Lin et al. (32)	Suspicion of brain involvement	To exclude nonconvulsive seizures/non-convulsive status epilepticus as a potential etiology of altered mental status, to monitor for continuing subclinical seizures after witnessed clinical seizures in patients; for monitoring the response to therapy for seizures, monitoring sedation levels, or for prognostication in the others
Louis et al. (33)	Suspicion of brain involvement	Seizure-like events n=5, altered mental status n=17
Marcic et al. (34)	Suspicion of brain involvement	Non-specific neurological symptoms such as headache, loss of sense of smell and taste, dizziness, and weakness
Pasini et al. (35)	Suspicion of brain involvement	Suspected COVID-19 related encephalopathy
Pastor et al. (36)	Suspicion of brain involvement	Clinical alterations of awareness or cognitive state n=20
Pellinen et al. (37)	Suspicion of brain involvement	Seizure-like events n=42, persistent encephalopathy n=72, seizure n=10, n=25 paroxysmal activity, n=11 prognostication after cardiac arrest
Petrescu et al. (38)	Suspicion of brain involvement	Delayed/inadequate awakens n=8, dysexecutive syndrome n=2, confusion n=9, fluctuating alertness n=10, myoclonus n=1, seizure n=3, unreactive mydriasis n=1, cardiac arrest n=1, nystagmus n=1
Saez-Landete et al. (39)	Suspicion of brain involvement	Indications for EEG studies included confusion, agitation, and disorientation in n=5, suspicious of epileptic seizures with disorientation and aggressiveness in n=1
Santos da Lima et al. (40)	Suspicion of brain involvement	Indications for EEG studies included evaluation of unexplained encephalopathy and suspicious of seizures
Skorin et al. (41)	Suspicion of brain involvement	Unexplained loss of consciousness without major abnormalities on blood tests and/or neuroimaging. Seizures or suspicious events were also indication
Sonkaya et al. (4)	Exploratory reasons	**/**
Waters et al. (42)	Suspicion of brain involvement	Hyperkinetic movements *n* = 30, altered mental status *n* = 22, persistent coma *n* = 23, prognostication after cardiac arrest and other reasons *n* = 4

*EEG, electroencephalography; COVID-19, coronavirus disease 2019*.

### Quality Assessment

Quality assessment of the included non-ICU studies is reported in Supplementary Table 5. Of the 21 articles included for review, two of them were rated to be of low quality, twelve of medium quality, and seven of high quality.

### Electroencephalogram

**Figure 2:**
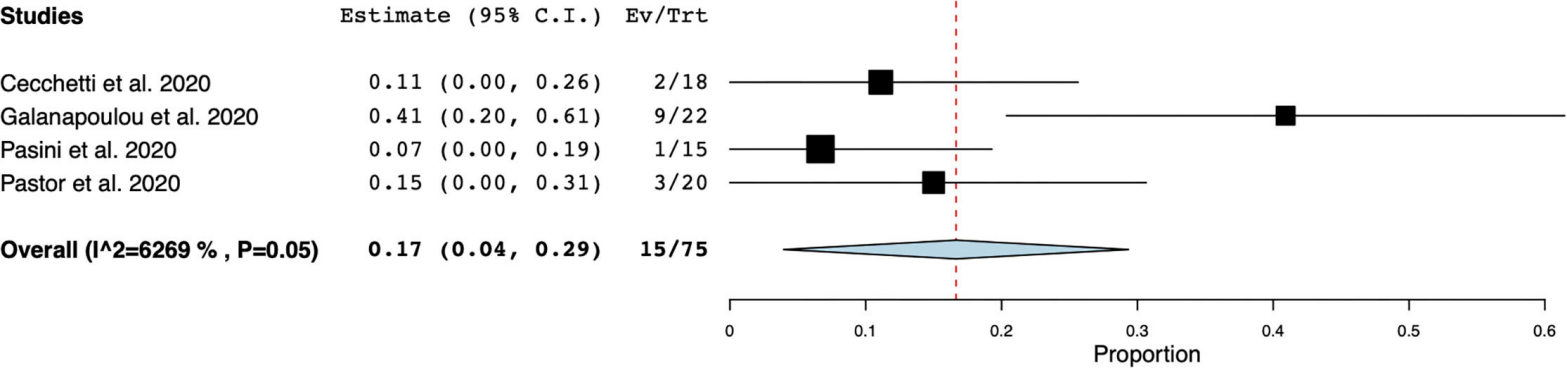
Forest plot of electroencephalographic seizures (ESz). ESz were found in 15/75 non-ICU patients, with an estimate pooled prevalence of ESz of 0.17 [95% Confidence Interval (CI) 0.04–0.29, standard error (SE) 0.06], τ^2^ 0.01, Q (df = 3) 8.04, *p* < 0.05, I^2^ = 62.69.

**Figure 3:**
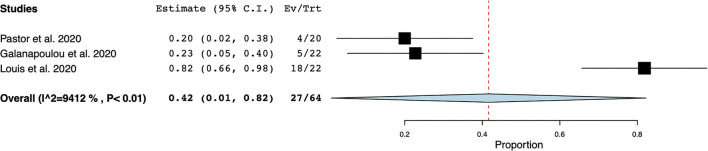
Forest plot of periodic and rhythmic patterns (RPPs) in non-ICU patients. RPPs was found in 27/64 patients with a pooled prevalence of 0.42 (95% Confidence Interval (CI) 0.01–0.82, SE 0.21, *p* < 0.04), τ^2^ 0.12, Q (df = 2) 34.02, *p* < 0.01, I^2^ = 94.12.

**Figure 4:**
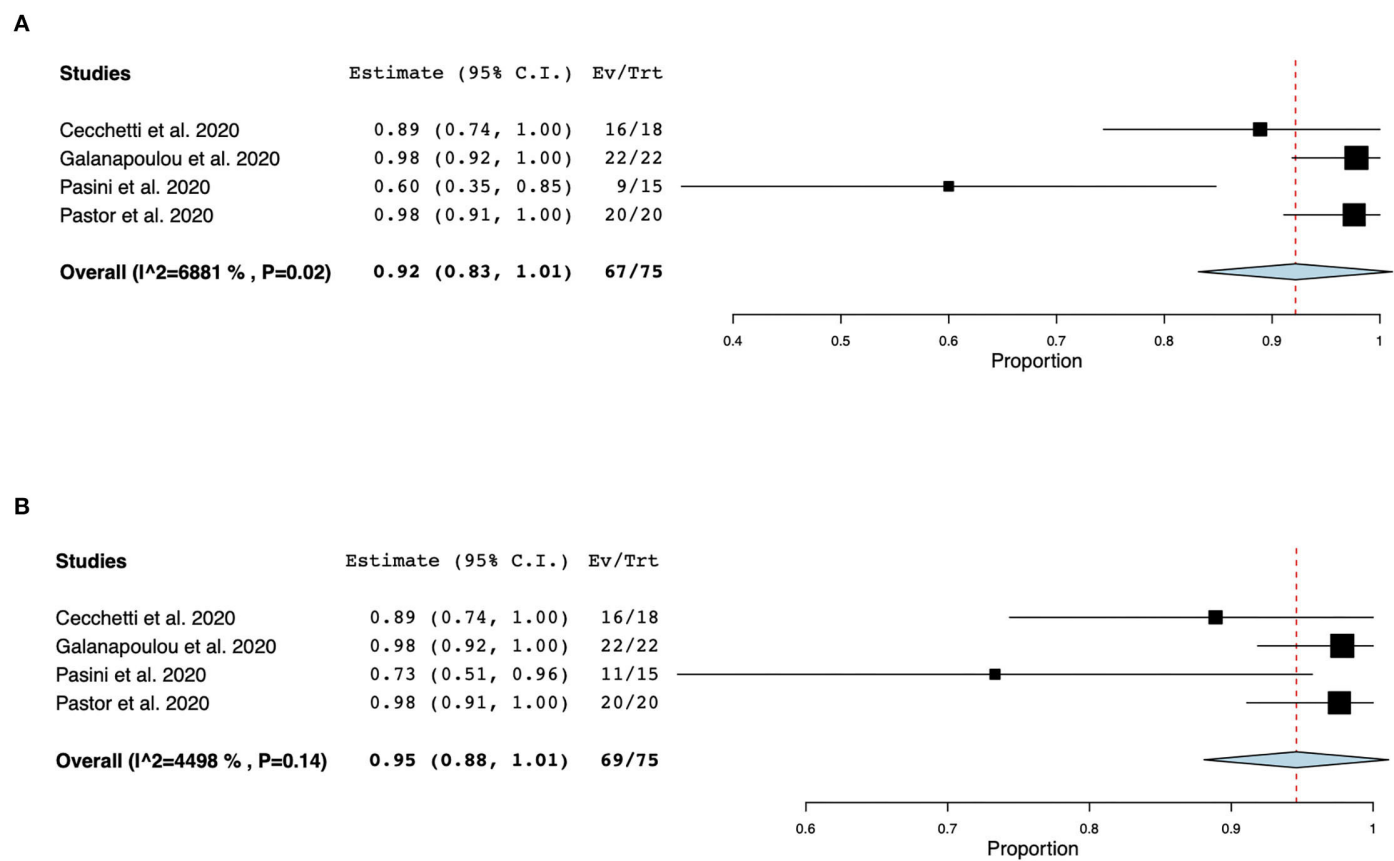
Forest plots of slow background (theta and delta) and abnormal background abnormalities. The pooled prevalence of slow background abnormalities was 0.92 (95% CI 0.83–1.01, SE 0.05, *p* < 0.01), τ^2^ 0.01, Q (df = 3) 9.62, *p* < 0.02, I^2^ = 68.81 **(A)**. The pooled prevalence of abnormal background in non-ICU COVID-19 patients was 0.95 (95%CI 0.88–1.09, SE 0.03, *p* < 0.01), τ^2^ 0.0, Q (df = 3) 5.45, *p* = 0.14, I^2^ 44.98 **(B)**.

EEG was the most frequently reported neuromonitoring modality during the COVID-19 pandemic in the non-ICU setting. The time from hospital-admission/ symptoms to EEG was highly variable and was not reported in most cases. Reasons for monitoring patients with EEG were (1) to identify EEG features in non-ICU patients with COVID-19, (2) to investigate the prevalence of seizures and other EEG abnormalities, and (3) to research neurological manifestations in non-ICU patients with COVID-19, (Supplementary Table 4). Electroencephalographic seizures (ESz) were found in 15 out of 75 patients investigated for EEG seizures, with an estimated pooled prevalence of ESz of.17 (95% CI 0.04–.29, I^2^ = 62.69%) in non-ICU patients with COVID-19, (Figure 2). EEG with periodic and rhythmic patterns (RPPs) was found in 21 out of 64 non-ICU patients with a pooled prevalence of.42 (95%CI 0.01–0.82, I^2^ = 94.12%), (Figure 3). The pooled prevalence of slow background abnormalities (theta and delta) in non-ICU patients was 0.92 (95%CI 0.83–1.01, I^2^ = 68.81%). The pooled prevalence of abnormal background in non-ICU patients was 0.95 (95%CI 0.88–1.09, I^2^ = 44.98%), (Figures 4A,B), respectively.

### Transcranial Doppler

Two studies regarding TCD monitoring in non-ICU patients with COVID-19 were found. Marcic et al. (34) reported the cerebral blood flow velocities (FVs) of non-ICU patients with COVID-19, while Sonkaya et al. (4) compared TCD monitoring in healthy volunteers and non-ICU patients with COVID-19, thus concluding that mean FV in the middle cerebral artery was higher in patients with COVID-19 than controls (*p* = 0.001), with a decreased vasomotor reactivity in those with COVID-19 (*p* = 0.001).

### Pupillometry

Karahan et al. (30) detected alteration in the pupillary diameter and reactivity in patients admitted in a non-ICU ward. Photopic and scotopic diameters were significantly higher in patients with COVID-19 than non-COVID-19 patients (*p* = 0.04 and *p* = 0.002), as well as resting pupillary diameter and pupil contraction velocity (*9* = 0.04 and *p* = 0.02, respectively). Pupil dilatation latency and contraction duration were lower in patients with COVID-19 than non-COVID-19 patients (*p* = 0.01 and *p* = 0.008, respectively). Ballavia et al. (25) evaluated autonomic dysfunction using an automated pupillometry in 20 non-critically ill patients vs. controls. The COVID-19 group presented higher values of pupillary dilatation velocities, autonomic dysfunction, and baseline pupillary diameter than the control group.

### Other Neuromonitoring Tools

No studies on NIRS and ONSD in the non-ICU population were found.

## Discussion

The main results of this systematic review and prevalence meta-analysis of abnormal findings detected using neuromonitoring tools in patients with COVID-19 outside the ICU can be summarized as follow: (1) non-invasive multimodal neuromonitoring tools are scarcely applied in non-critical care settings, and patients with no primary brain injury such as COVID-19, (2) with a pooled prevalence of electroencephalographic findings, including ESz, RPPs, slow background, and abnormal background [0.17 (95% CI 0.04–0.29), 0.42 (95%CI 0.01–0.82), 0.92 (95%CI 0.83–1.01), and 0.95 (95%CI 0.88–1.09), respectively] are relevant in the non-ICU COVID-19 population; (3) altered cerebral blood flow and vasomotor reactivity can be detected by TCD in patients with COVID-19 outside the ICU, (4) automated pupillometry can help with the identification of altered pupillary diameter and reactivity in non-critically ill patients with COVID-19.

Patients affected by COVID-19 are at high risk of neurological sequelae, both during hospital stay (43, 44) and at long-term follow-up (45). In critically ill patients with COVID-19, one of the most frequently reported neurological complications was delirium, with a high prevalence of acute and prolonged brain dysfunction (46). Stroke was more prevalent among patients admitted to the ICU vs. those in non-ICU (2.8 vs. 1.3%) (45). In a critically ill setting during the COVID-19 pandemic, new neurological complications occurred with high incidence and severity, which can be justified by the frequent need of life-sustaining therapies and the potential for multiple organ failure. Indeed, patients with COVID-19 who underwent extracorporeal membrane oxygenation manifested severe neurologic events in 25% of cases (47). Although the incidence and severity of neurological manifestations in patients with COVID-19 seemed to be predominant in the ICU setting, the literature confirmed that it was certainly crucial also in a non-critically ill setting. Indeed, neurological manifestations in hospitalized patients included myalgia (22%), ageusia (20%), anosmia (18%), headache (12%), dizziness (11%), acute encephalopathy (9%), and other minors, with potentially devasting consequences such as an impaired quality of life, residual disability at 6-months, impaired cognition, and persistence of anxiety and depression (48, 49).

Non-invasive neuromonitoring tools are increasingly applied in the non-primary brain injured population to identify those patients early who may have potential neurologic derangements (50–52). Each neuromonitoring tool can detect specific neurologic features. TCD allows the investigation of cerebral vessels' patency, cerebral blood flow, intracranial pressure, cerebral compliance, and new brain lesions; ONSD allows the identification of high intracranial pressure; NIRS detects changes in cerebral oxygenation; automated pupillometry easily detects pupillary changes and autonomic dysfunction, and EEG can identify irregularities of brain activity like seizures, background abnormalities, or slowing patterns (53). Although the use of neuromonitoring is becoming the standard of care even in patients who are not primarily brain injured in the ICU setting, their application in a non-ICU setting is still limited. In our study, we found no reports investigating neurological features *via* ONSD and NIRS outside the ICU, while data on TCD were limited to two studies from which it was impossible to calculate the prevalence of altered neuromonitoring features and only two studies on pupillometry.

Despite the limited application of neuromonitoring tools outside the ICU during the COVID-19 pandemic, EEG findings revealed various abnormalities among non-critically ill patients with COVID-19 who manifested new neurological symptoms. The most common finding was the presence of abnormal background activity, followed by slow background, rhythmic and periodic discharges, and electroencephalographic seizures. This may be explained by various factors: (1) patients with CVOID-19 might be at higher risk of hypoxic and metabolic changes responsible for encephalopathy (54), (2) after the virus enters the cells, a strong inflammatory response followed by cytokine storms may alter cerebral permeability and hemodynamic, thus favoring encephalopathy and multiple organ failure with potential for EEG alterations (55–57), (3) seizures, although a prevalence comparable to the non-COVID-19 population may be indicative of new neurological complications (58). To date, other systematic reviews and prevalence meta-analyses investigating EEG abnormalities in patients with COVID-19 have been published (59–62). However, none of them distinguished ICU from non-ICU patients, making the estimation of a real pooled prevalence in these two patient populations difficult. Hence, our prevalence meta-analysis is the first to investigate the prevalence of abnormal EEG findings in non-ICU patients only.

As inferred from the literature, TCD is more frequently applied in ICU than non-ICU settings (5, 6, 63–65). TCD is particularly useful for the detection of intracranial lesions, evaluating brain anatomy, and assessing cerebral hemodynamic at the bedside (66). TCD allows the assessment of non-invasive intracranial pressure to detect patients at risk of intracranial hypertension (67). It is also able to detect altered cerebral autoregulation that is associated with a poor outcome in many diseases and increases the risk of cerebral damage (68). In case of cerebral vasospasm, shunt, or micro-emboli, TCD may help to evaluate the constriction and patency of cerebral vessels (69). Similar to critically ill patients with COVID-19 (5, 70, 71), altered cerebral blood flow velocities, and vasomotor reactivity were detected by TCD in two studies in non-critically ill patients (4, 34). This can be explained by various mechanisms: (1) respiratory failure with altered oxygen and carbon dioxide exchange that may have altered the cerebral vessels' diameter and autoregulation (72–74) (2) the use of non-invasive respiratory devices that may have increased intraabdominal and intrathoracic pressures (75), both of which may impair cerebral hemodynamics (76), and (3) the potential of inflammation and cytokines to have induced sympathetic over activity with changes in systemic and cerebral hemodynamic and cerebral autoregulation *via* vasogenic edema with an altered permeability of the blood-brain barrier (55, 56). For all these reasons, the use of TCD at the bedside in non-critically ill patients with COVID-19 can be considered when suspicion of new neurological complications is high.

Lastly, altered pupillary diameter and reactivity were commonly found *via* automated pupillometry (30). In both studies, in contrast to the other neuromonitoring tools, the reason for neuromonitoring was mainly exploratory and not for the clinical suspicion of new neurological events (25, 30). Pupillary findings in patients with COVID-19 admitted to the ICU could be justified by the use of sedatives and analgesics, but in non-ICU patients with COVID-19 who were not sedated, this can be much easily explained by a status of autonomic impairment, orthostatic intolerance, and cognitive deceleration that is typical of this patient population (5, 30, 77). Among neurological manifestations, recent evidence of autonomic dysfunction has been reported as the long-COVID-19 syndrome but is lacking confirmatory data, especially in the context of non-critically ill patients (78, 79). During the first phase of an infection, sympathetic activity is predominant. On the contrary, parasympathetic activity acts primarily in the later stages of the infection to mitigate the systemic inflammatory response (80, 81). These results, despite coming from only two studies, suggest that dysautonomia could significantly contribute to the spectrum of COVID-19-related neurological disorders. The early recognition of dysautonomia could improve the in-hospital management of patients with COVID-19.

In summary, although the use of non-invasive multimodal neuromonitoring for the detection of such new neurological features is still limited to a few neuromonitoring tools and patients (with predominant use in the ICU setting), the prevalence of cerebral derangements in patients with COVID-19 outside the ICU is high when neurologically monitored. As non-invasive neuromonitoring tools are less expensive, quick, safe, and easily available at the bedside, they should be implemented for the detection of acute neurological features in patients with COVID-19 outside the ICU.

### Limitations

This systematic review/meta-analysis presents several limitations. Several of the studies included in our review were variably performed in a mixed ICU/non-ICU setting. Therefore, we excluded from the prevalence analysis the studies where it was impossible to distinguish ICU from non-ICU patients. A direct comparison between ICU and non-ICU patients was not possible due to the high heterogeneity of data and populations, the characteristics of patients, and the type of neuromonitoring tools. Additionally, we found a high heterogeneity among sample sizes (the largest was 197 and the smallest was 10), study design, and non-standardized neuromonitoring tools. Also, the indications for neuromonitoring as well as the ability of neuromonitoring tools to detect new neurological derangements were highly heterogeneous. Finally, our search was only focused on brain neuromonitoring, lacking investigation of peripheral nerve conduction studies and electromyography, which might merit further investigation.

## Conclusions

Neuromonitoring tools are non-invasive, less expensive, safe, and available at the bedside with great potential for both diagnosis and monitoring of patients with CVOID-19 at risk of brain derangements. However, non-invasive multimodal neuromonitoring tools are infrequently applied in non-critical care settings and non-primarily brain-injured patients with COVID-19. Given the incidence of neurological complications in this patient population and the potential long-term effects of such injuries, it is likely that neuromonitoring will have an increasingly important role in the future. Clinicians should have a good understanding of these tools to facilitate their use and appreciate their impact on clinical practice as preventing measures for better patient neurological outcomes. In the same way, researchers should develop and implement clinical studies to enhance and validate the role of neuromonitoring in patients with no primary brain injury.

## Data Availability Statement

The raw data supporting the conclusions of this article will be made available by the authors, without undue reservation.

## Author Contributions

DB, CR, LP, and S-MC: study design. DB and LP: study search, study selection, and data extraction. DB and CR: assessment of methodological quality. DB: statistical analysis and manuscript preparation. CR, S-MC, LP, SH, JF, GW, RA, JB, DBP, FT, JS, RB, GL, and JF: critical revision of the manuscript. All authors have read and approved the submitted manuscript.

## Conflict of Interest

The authors declare that the research was conducted in the absence of any commercial or financial relationships that could be construed as a potential conflict of interest.

## Publisher's Note

All claims expressed in this article are solely those of the authors and do not necessarily represent those of their affiliated organizations, or those of the publisher, the editors and the reviewers. Any product that may be evaluated in this article, or claim that may be made by its manufacturer, is not guaranteed or endorsed by the publisher.
