# Supplementary material for: Non-Invasive Multimodal Neuromonitoring in Non-Critically Ill Hospitalized Adult Patients With COVID-19: A Systematic Review and Meta-Analysis
Source: Front Neurol. 2022 Apr 14;13:814405. doi: 10.3389/fneur.2022.814405 (PMC9047047; doi:10.3389/fneur.2022.814405)
Supplement: Supplementary file 1 [file Data_Sheet_1.docx]

**Supplementary Material non—ICU**

**Table of contents:**

Supplementary Material Table 1: PRISMA checklist [page 2-3]

Supplementary Material Item 1: full search strategy [page 4-5]

Supplementary Material Table 2: characteristics of the included studies in Hospital (non-ICU only or mixed ICU) setting [page 6-10]

Supplementary Material Table 3: characteristics of EEG findings of the studies included in non-ICU setting [page 11-12]

Supplementary Item 2: quality assessment non-ICU [page 13-14]

Supplementary Material Table 4: quality assessment non-ICU [page 15]

References [page 16-18]

**Supplementary Material Table 1. PRISMA checklist**

| **Section/topic** | **#** | **Checklist item** | **Reported on page** |
| --- | --- | --- | --- |
| **TITLE** | | |  |
| Title | 1 | Identify the report as a systematic review, meta-analysis, or both. | 1 |
| **ABSTRACT** | | |  |
| Structured summary | 2 | Provide a structured summary including, as applicable: background; objectives; data sources; study eligibility criteria, participants, and interventions; study appraisal and synthesis methods; results; limitations; conclusions and implications of key findings; systematic review registration number. | 4 |
| **INTRODUCTION** | | |  |
| Rationale | 3 | Describe the rationale for the review in the context of what is already known. | 5 |
| Objectives | 4 | Provide an explicit statement of questions being addressed with reference to participants, interventions, comparisons, outcomes, and study design (PICOS). | 5 |
| **METHODS** | | |  |
| Protocol and registration | 5 | Indicate if a review protocol exists, if and where it can be accessed (e.g., Web address), and, if available, provide registration information including registration number. | 6 |
| Eligibility criteria | 6 | Specify study characteristics (e.g., PICOS, length of follow-up) and report characteristics (e.g., years considered, language, publication status) used as criteria for eligibility, giving rationale. | 6 |
| Information sources | 7 | Describe all information sources (e.g., databases with dates of coverage, contact with study authors to identify additional studies) in the search and date last searched. | 6 |
| Search | 8 | Present full electronic search strategy for at least one database, including any limits used, such that it could be repeated. | Sup. mat |
| Study selection | 9 | State the process for selecting studies (i.e., screening, eligibility, included in systematic review, and, if applicable, included in the meta-analysis). | 6 |
| Data collection process | 10 | Describe method of data extraction from reports (e.g., piloted forms, independently, in duplicate) and any processes for obtaining and confirming data from investigators. | 7 |
| Data items | 11 | List and define all variables for which data were sought (e.g., PICOS, funding sources) and any assumptions and simplifications made. | 7 |
| Risk of bias in individual studies | 12 | Describe methods used for assessing risk of bias of individual studies (including specification of whether this was done at the study or outcome level), and how this information is to be used in any data synthesis. | 7 |
| Summary measures | 13 | State the principal summary measures (e.g., risk ratio, difference in means). | 6/7 |
| Synthesis of results | 14 | Describe the methods of handling data and combining results of studies, if done, including measures of consistency (e.g., I^2^) for each meta-analysis. | 6/7 |
| Risk of bias across studies | 15 | Specify any assessment of risk of bias that may affect the cumulative evidence (e.g., publication bias, selective reporting within studies). | 7 |
| Additional analyses | 16 | Describe methods of additional analyses (e.g., sensitivity or subgroup analyses, meta-regression), if done, indicating which were pre-specified. | 7 |
| **RESULTS** | | |  |
| Study selection | 17 | Give numbers of studies screened, assessed for eligibility, and included in the review, with reasons for exclusions at each stage, ideally with a flow diagram. | 8 |
| Study characteristics | 18 | For each study, present characteristics for which data were extracted (e.g., study size, PICOS, follow-up period) and provide the citations. | Table 1 |
| Risk of bias within studies | 19 | Present data on risk of bias of each study and, if available, any outcome level assessment (see item 12). | 8 |
| Results of individual studies | 20 | For all outcomes considered (benefits or harms), present, for each study: (a) simple summary data for each intervention group (b) effect estimates and confidence intervals, ideally with a forest plot. | 8-14 |
| Synthesis of results | 21 | Present results of each meta-analysis done, including confidence intervals and measures of consistency. | 8-9 |
| Risk of bias across studies | 22 | Present results of any assessment of risk of bias across studies (see Item 15). | Sup. mat |
| Additional analysis | 23 | Give results of additional analyses, if done (e.g., sensitivity or subgroup analyses, meta-regression [see Item 16]). | N/A |
| **DISCUSSION** | | |  |
| Summary of evidence | 24 | Summarize the main findings including the strength of evidence for each main outcome; consider their relevance to key groups (e.g., healthcare providers, users, and policy makers). | 15-17 |
| Limitations | 25 | Discuss limitations at study and outcome level (e.g., risk of bias), and at review-level (e.g., incomplete retrieval of identified research, reporting bias). | 16-17 |
| Conclusions | 26 | Provide a general interpretation of the results in the context of other evidence, and implications for future research. | 17 |
| **FUNDING** | | |  |
| Funding | 27 | Describe sources of funding for the systematic review and other support (e.g., supply of data); role of funders for the systematic review. | 18 |

**Supplementary Material Item 1. Full search strategy**

**PubMed**

(coronavirus disease OR COVID-19 OR coronavirus disease 2019 OR SARS-CoV-2 OR covid or coronavirus) AND (electroencephalography OR electroencephalogram OR BIS OR bispectral index OR EEG monitoring OR EEG OR neuromonitor* OR near infrared spectroscopy OR NIRS OR transcranial doppler OR TCD OR TCCS OR ONSD OR optic nerve sheath diameter OR optic nerve OR pupillometer OR pupillometry OR intracranial pressure OR ICP OR cerebral compliance OR flow velocities)

**MEDLINE**

("coronavirus disease" OR "COVID-19" OR "coronavirus disease 2019" OR "SARS-CoV-2" OR "covid" or "coronavirus") AND ("electroencephalography" OR "electroencephalogram" OR "BIS" OR "bispectral index" OR "EEG monitoring" OR "EEG" OR "neuromonitor*" OR "near infrared spectroscopy" OR "NIRS" OR "transcranial doppler" OR "TCD" OR "TCCS" OR "ONSD" OR "optic nerve sheath diameter" OR "optic nerve" OR "pupillometer" OR "pupillometry" OR "intracranial pressure" OR "ICP" OR "cerebral compliance" OR "flow velocities")

**Scopus**

( "coronavirus* disease*"  OR  "COVID-19"  OR  "SARS-CoV-2"  OR  "covid*" )  **AND**  ( "electroencephalog*"  OR  "EEG*"  OR  "BIS"  OR  "bispectral* index*"  OR  "EEG* monitoring*"  OR  "neuromonitor*"  OR  "near* infrared* spectroscopy*"  OR  "NIRS"  OR  "transcranial*doppler*"  OR  "TCD"  OR  "TCCS"  OR  "ONSD"  OR  "optic* nerve* sheath* diameter*"  OR  "optic* nerve*"  OR  "pupillomet*"  OR  "intracranial* pressure*"  OR  "ICP*"  OR  "cerebral* compliance*"  OR  "flow* velocit*"  OR  "transcranial* color* sonograp*"  OR  "brain* sonograph*"  OR  "brain* ultrasound*" ) AND  ( LIMIT-TO ( DOCTYPE ,  "ar" )  OR  LIMIT-TO ( DOCTYPE ,  "re" )  OR  LIMIT-TO ( DOCTYPE ,  "le" ) )  AND  ( LIMIT TO ( SUBJAREA ,  "MEDI" ) )

**EMBASE**

('coronavirus disease' OR (('coronavirus'/exp OR coronavirus) AND ('disease'/exp OR disease)) OR 'covid 19'/exp OR 'covid 19' OR 'coronavirus disease 2019'/exp OR 'coronavirus disease 2019' OR (('coronavirus'/exp OR coronavirus) AND ('disease'/exp OR disease) AND 2019) OR 'sars cov 2'/exp OR 'sars cov 2' OR 'covid'/exp OR covid OR 'coronavirus'/exp OR coronavirus) AND ('electroencephalography'/exp OR electroencephalography OR 'electroencephalogram'/exp OR electroencephalogram OR bis OR 'bispectral index'/exp OR 'bispectral index' OR (bispectral AND index) OR 'eeg monitoring'/exp OR 'eeg monitoring' OR (('eeg'/exp OR eeg) AND ('monitoring'/exp OR monitoring)) OR 'eeg'/exp OR eeg OR neuromonitor* OR 'near infrared spectroscopy'/exp OR 'near infrared spectroscopy' OR (near AND ('infrared'/exp OR infrared) AND ('spectroscopy'/exp OR spectroscopy)) OR nirs OR 'transcranial doppler'/exp OR 'transcranial doppler' OR (transcranial AND doppler) OR tcd OR tccs OR onsd OR 'optic nerve sheath diameter'/exp OR 'optic nerve sheath diameter' OR (optic AND ('nerve'/exp OR nerve) AND sheath AND diameter) OR 'optic nerve'/exp OR 'optic nerve' OR (optic AND ('nerve'/exp OR nerve)) OR 'pupillometer'/exp OR pupillometer OR 'pupillometry'/exp OR pupillometry OR 'intracranial pressure'/exp OR 'intracranial pressure' OR (intracranial AND ('pressure'/exp OR pressure)) OR icp OR 'cerebral compliance' OR (cerebral AND ('compliance'/exp OR compliance)) OR 'flow velocities' OR (('flow'/exp OR flow) AND velocities))

**Cochrane**

ID Search Hits

#1 MeSH descriptor: [COVID-19] explode all trees

#2 MeSH descriptor: [Ultrasonography, Doppler, Transcranial] explode all trees

#3 MeSH descriptor: [Electroencephalography] explode all trees

#4 MeSH descriptor: [Optic Nerve Sheath] explode all trees

#5 MeSH descriptor: [Pupillometry] explode all trees

#6 MeSH descriptor: [Bispectral index] explode all trees

#7 MeSH descriptor: [Spectroscopy, Near-Infrared] explode all trees

**Supplementary Material Table 2**

| First  Author | Study design | Country | Total, Male/  Female | Age | Neurological  History | New symptoms/ neurological findings | Mortality | Sedation during nm | Type of nm | Subtype  Eeg only | Time to nm | Primary  Outcome | Hospital/ ICU |
| --- | --- | --- | --- | --- | --- | --- | --- | --- | --- | --- | --- | --- | --- |
| Ayub et al. (1) | RO | USA | 37, 27/10 | 66 (59.5-76.3) | Seizures n=1, CNS disorders n=8, psychiatric history n=16, altered mental status n=28, anosmia n=4 | Abnormal neuroimaging n= 9 (n=3 AIS, n=3 ICH, 1 meningitis in HIV), acute CNS injury n=9.  Convulsion n=11, altered mental status n=24 | n=10 | n=27 | EEG | EEG according to the 10–20 international system | 17.5 (8.5-27.5) days from symptoms | To test the hypothesis that there is a moderate rate of epileptiform discharges in patients with SARS-CoV-2, but that they are most common in patients with preexisting, rather than new, brain pathology | n=28 ICU and n=9 hospital |
| Bellavia et al. (2) | PO | Italy | 20, 14/13 | 54.3 (16.43) | NR | NR | NR | n=0 | Pupillometry | / | NR | To assess the differences in pupillary response, and autonomic activity between COVID-19 and nonCOVID-19 | n=20 hospital |
| Besnard et al. (3) | RO | France | NR | NR | NR | NR | NR | n=4 | EEG | NR | NR | To retrospectively analyze and reporte the EEG patterns of 42 COVID-19 patients | ICU and hospital |
| Cecchetti et al. (4) | CS | Italy | 18, 11/7 | Group Normal 62.4 (15.9), group Moderate 70.4 (8.8), group severe 64.3 (4.5) | n=1 remote IPH? n=1 remote ischemic lesion | PRES n=1, glioblastoma n=1, brain metastasis n=1, SDH n=1, demyelinating lesion n=1 | NR | NR | EEG | cEEG | NR | To investigate EEG abnormalities in COVID-19 patients. | n=18 hospital |
| Corazza et al. (5) | RO | Brazil | 28, 11/17 | 58 (18-86) | n=0 | Altered mental status n=19, seizures n=8 | n=16 | n=6 | EEG | cEEG according to the 10-20 international system | NR | To report EEG finding in COVID-19 | ICU and medical ward |
| Galanapoulou et al. (6) | CS | USA | 22, 14/8 | 63.23 (11.9) | Epilepsy n=4, neurological disorders n=7, psychiatric disorders n=5 | Altered mental status n=20, seizure like events n=14, confusion n=1, gaze deviation n=1, SAH n=1, subdural hematoma n=1, subcortical hyperintensity at MRI n=1 | NR | n=14 | EEG | 8 channels EEG n=20, cEEG n=7, EEG n=4 | NR | To investigate EEG abnormalities in COVID-19 patients. | n=22 hospital |
| Karahan et al. (7) | PO | Turkey | 60, 35/25 | 37.65 (7.06) | NR | NR | NR | NR | Pupillometry | / | 30 days after recovery | To investigate the detection of autonomic dysfunction by dynamic and static pupillary responses in patients recovering from COVID-19 with the aid of automatic pupillometry. | n=60 patients hospital and n=40 controls |
| Lambrecq et al. (8) | PO | France | 78, 57/21 | 61 (12) | NR | Anosmia n=12, ageusia n=9, headhache n=8, delirium n=44, seizures n=10, dizziness n=2, visual disturbances 1/75, oculomotor disorders n=6, movement disorders n=15, sleep disorders n=4, language disorders n=16, disorder of consciousness n=28, brainstem impairment n=7, cerebellar syndrome n=5, cognitive disorders n=36, frontal syndrome n=15 | n=7 | n=4 | EEG | EEG over 20 min using a 8-21-channel. | 29.4 (21.3) days from symptoms onset | To investigate clinical, biological, and brain magnetic resonance imaging (MRI) findings in association with electroencephalographic (EEG) features for patients with COVID-19, and to better refine the features of COVID-19-related encephalopathy. | n=41 ICU, n=37 hospital |
| Lin et al. (9) | RO | USA | 197, 118/79 | 65 (57-73) | n=111 previous history of CNS disorders: epilepsy n=32, CNS disorders included epilepsy n=67 | Seizures n=38, altered mental status n=120 | n=73 | NR | cEEG | 21-channel cEEG electrodes recording using the international modified 10–20 system. | 7 (2-18) days from hospitalization | Prevalence and risk factors for electrographic seizures and other cEEG patterns in patients with COVID‐19 | Hospital and ICU (% NS, n=161 mechanically ventilated during hospital stay) |
| Louis et al. (10) | RO | USA | 22, 14/8 | 66.5 (11.2) | Epilepsy n=2, stroke n=1, headache n=1, traumatic brain injury n=1, spinal stenosis n=1 | Seizures n=2 | n=6 | n=14 | EEG, cEEG | cEEG 19 patients with 21-electrodes for at least 24 h, and 3 underwent routine EEG (<1 h) with 21 electrodes. | NR | To investigate EEG abnormalities in COVID-19 patients. | n=22 hospital |
| Marcic et al. (11) | PO | Croatia | 25, 16/9 | 46.6 (8.5) | NR | Dysgeusia n=14, n=10 dizziness, n=17 headache, n=18 fatigue and myalgia | NR | NR | TCD | / | NR | To investigate and describe cerebral hemodynamic in covid-19 and non-covid-19 | n=25 hospital |
| Pasini et al. (12) | CS | Italy | 15, 6/9 | 64.6 (47-79) | Cognitive decline n=2, limbic encephalitis n=1, frontal metastasis n=1 | Confusion n=11, aphasia n=1, impaired consciousness n=4. | NR | NR | EEG | NR | 8 days from neurological symptoms | To present the EEG characteristics of 15 patients experiencing varying degrees of cerebral dysfunction | n=15 hospital |
| Pastor et al. (13) | RO | Spain | 20, 17/3 | 63.9 (2.7) | n=0 | Stuporous n=8, confused n=10 | NR | NR | EEG | cEEG according to the 10–20 international system, qEEG | NR | To investigate EEG abnormalities in COVID-19 patients. | n=20 hospital |
| Pati et al. (14) | CS | USA | 10, 5/5 | 61.3 (good outcome 56.2 (48-68), poor outcome 66.4 (47-85) | NR | NR | n=4 | NR | cEEG, qEEG | cEEG EEG according to the 10–20 international system for 48 hours | NR | To investigate EEG abnormalities in COVID-19 patients. | n=10 hospital |
| Pellinen et al. (15) | RO | USA | 111, 79/32 | 64 (56-73) | Epilepsy n=13, ischemic stroke n=23, other brain disorders n=19 (ICH n=4, dementia n=4, disability n=3, brain tumor n=2, traumatic brain injury n=2, Parkinson n=2, vascular malformations n=1, tuberous sclerosis complex n=1, herpes encephalitis n=1) | AIS n=18, ICH n=15, ischemia/hemorrhage n=3, cerebral edema n=6, diffuse leukoencephalopathy n=4 | n=49 | n=67 | EEG, cEEG | cEEG 21-channel EEG with 21-electrodes 10-20 system for at least 24 hours. In some cases, EEG was recorded using a portable rapid EEG system comprising a reduced 8-bipolar channel montage for 0.5-12 hours. | 10.8 (17.3) days from hospitalization | To determine the prevalence of EEG abnormalities and identify clinical, laboratory, and imaging factors associated with seizures and epileptogenic abnormalities in this population. | n=85 ICU and n=26 hospital |
| Petrescu et al. (16) | RO | France | 36, 29/7 | 69.68 (13.18) | Dementia n=9, Parkinson n=1, hydrocephalus n=1, epilepsy n=1, chronic subdural hematoma n=2, stroke n=2, memory impairment n=1, neuropathy n=1 | Delayed/inadequate awakeness n=8, diexecutive syndrome n=2, confusion n=, fluctuating alertness n=10, myoclonus n=, seizure n=3, unreactive mydriasis n=1, cardiac arrest n=1, nystagmus n=1 | n=2 | n=13 | EEG | EEG according to the 10–20 international system for 20 min | NR | To investigate EEG abnormalities in COVID-19 patients. | n=18 ICU and n=22 hospital (40 EEGs in 36 patients) |
| Saez-Landete et al. (17) | RO | Spain | 15, 14/1 | 68.5 (11.2) | Mild cognitive impairment n=1, lacunar ischemic infarct n=1 | Aphasia n=1, altered consciousness n=13, myoclonus n=1, flaccid tetra paresis n=1, hemiparesis n=1, suspicious of seizures n=2 | n=4 | n=5 | EEG | EEG according to the 10–20 international system, continuous |  | To investigate EEG abnormalities in COVID-19 | 15 (n=6 hospital, n=9 ICU) |
| Santos de lima et al. (18) | RO | USA | 32, 18/14 | 61.9 (17.8) | Dandy-Walker n=1, Alzheimer n=1 | Seizures n=4, subdural hematoma n=1 | n=6 | NR | cEEG | cEEG electrodes recording using the international modified 10–20 system plus supplementary subtemporal electrodes (F9, T9, M1, F10, T10 and M2); in three cases, early in the pandemic, a limited montage consisting of eight channels was used. | NR | To determine the prevalence of epileptiform activity and to assess the risk of seizures in patients with COVID-19 | ICU and hospital |
| Skorin et al. (19) | PO | Chile | 62, 34/28 | 59.7 (17.8) | CNS disorders n=24 | AIS n=3, parenchymal hematomas n=2, subdural hematoma n=2, subarachnoid hemorrhage n=2, cerebral venous thrombosis n=2, brain edema n=2, PRES n=1 | n=17 | n=38 | EEG, cEEG | Portable 87/94 EEG (30 min-1 h) and cEEG monitoring (> 12 h) in n=7 EEG in n=62 patients with a Cadwell’s Easy II EEG system at UC and a Natus NeuroWorks at HSR with 21 electrodes international modified 10–20 system. | 11.9 (9.2) days from starting symptoms | To describe the most prevalent electroencephalographic findings in COVID-19 hospitalized patients, and to determine possible predictors of mortality including EEG and clinical variables | ICU n=37 and hospital n=25 |
| Sonkaya et al. (20) | RO | Turkey | 20, 13/7 | 50.15 (19.07) | NR | NR | NR | NR | TCD | / | NR | To investigate the cerebrovascular hemodynamics in patients with COVID-19 via TCD and to evaluate the vasomotor reactivity capacity with the breath-holding index. | n=20 hospital COVID-19, n=20 non-COVID-19 |
| Waters et al. (21) | RO | USA | 79, 54/25 | 64 (57-70) | n=7 history of seizures, chronic brain disease n=24 | Acute brain injury n=27, hypoxic ischemic injury n=4, AIS n=11, acute ICH 12, PRES n=1, worsening vasogenic edema n=1 | n=21 | NR | cEEG | 21-channel cEEG electrodes recording using the international modified 10–20 system. | NR | To describe the results of 257 EEG days across 79 patients as well as to identify the incidence of, and risk factors for, detecting seizures on cEEG | n=64 ICU, n=15 hospital |

Table Legend: ICU, intensive care unit; CS, case series; CR, case report; RO, retrospective observational; PO, prospective observational; EEG, electroencephalogram; cEEG, continuous electroencephalogram; CNS, central nervous system; AIS, acute ischemic stroke; ICH, intracranial hemorrhage; PRES, reversible posterior encephalopathy syndrome; NR, not reported; ECMO, extracorporeal membrane oxygenation; Data expressed as mean (standard deviation) OR median (interquartile range)

**Supplementary Material Table 3**

| **First Author** | **N. of patients** | **Type of EEG abnormality** | **Diagnostic and prognostic value** |
| --- | --- | --- | --- |
| **Ayub et al.** | 37 | A background G delta/theta slowing n=31, D alpha n=6, D beta n=5, F delta slowing n=2, F theta slowing n=1, BS n=5, absent posterior dominant rhythm n=34, moderately low voltage n=4, U n=1  RPPs (G PDs 12/37, stimulus-induced rhythmic or ictal PDs n=3, G RDA n=5, L RDA n=1  ESz (NCSE n=1)  Sporadic discharges BIRDS (F n=1, Mf n=6, G n=17) | NR |
| **Besnard et al.** | 42 | A slight deceleration n=9, not specific A n=8  Normal n=12  ESz F/G n=4 (ECSE n=1)  RPPs (RDA/PDs/SW) n=9 | NR |
| **Cecchetti et al.** | 18 | Normal n=5  G slow waves n=16 (F anterior slowing n=10)  ESz n=2 | NR |
| **Corazza et al.** | 28 | Background theta n=10, triphasic waves n=2, diffuse attenuation n=7, posterior activity n=3, BS n=1  ESz n=1 | Not found association between EEG and complications |
| **Galanapoulou et al.** | 22 | Slowing background n=22 (BI 22, F 5), no AP gradient n=17, S n=18, A n=3, BS n=1  ESz n=9 (frontal 8, temporal 2)  RPPs n=5 (G n=3, BI n=1, L n=1) |  |
| **Lambrecq et al.** | 78 | A (n=69), PDs n=6, ESz n=4  A background (n=63): without PD/RDA/slow waves n=12, frontal slow waves n=47 (including an encephalopathy pattern (n=23), and frontal slow waves (n=24) | Patients with focal frontal abnormalities had a less-frequent total recovery of neurologic symptoms at hospital discharge than those with other abnormalities (1 of 10 [10%] vs 4 of 7 [57%]; P = .05). No EEG pattern was associated with death at hospital discharge. |
| **Lin et al.** | 197 | ECSz n=19 (n=11 NCSE, F n=12, Mf/BI n=3, G n=4) | ECSz was significantly associated with mortality (HR 4.07, 95%CI 1.44–11.51, p < 0.01). Death occurred in 63/178 (35.4%) patients without ECSz versus 10/19 (52.7%) patients with ECSz. |
| **Louis et al.** | 22 | ESz n=5  Posterior dominant rhythm n=11,  RPPs: GPDs n=7, GRDA n=11 | Among the 22 patients in the study population, there were no statistically significant differences between patients who expired in the hospital and those who remain alive with respect to the presence of epileptic EEG findings (p = 0.585), PDR (p = 0.635), GPD (0.616), or GRDA (p = 0.149). |
| **Pasini et al.** | 36 | A slow background activity n=13 (n=5 theta, n=4 theta/delta, F delta/theta n=3, FRDA n=1) | NR |
| **Pastor et al.** |  | Slow background n=20  RPPs (L PDs 1/20, SW 3/20) | NR |
| **Pellinen et al.** | 111 | Normal n=5 patients, abnormal G slowing n=17, moderate G slowing n=60, severe G slowing n=29, F slowing n=27  RPPs: PDs/RDA (LRDA n=7, GRDA n=4, both n=2, LDPs n=3, GPDs n=11)  ESz n=35 (focal n=12, Mf n=6, G n=5), NCSE n=2 | There was no observed relationship between epileptiform EEG and in-hospital mortality in adult patients without cardiac arrest (11 of 28 patients with an epileptiform EEG subsequently died and 17 of 58 without subsequently died: OR 0.91, 95% CI 0.33-2.4) |
| **Petrescu et al.** | 36 | Slow background activity (theta), preserved antero-posterior gradient and R n=19  Slow background activity (theta), preserved antero-posterior gradient and R sporadic slow waves diphasic/triphasic n=4  Normal n=4  PRRs (RDA n=6, G PDs n=3, Mf PDs n=2, C RDA UR n=1, D RDA UR n=1, C G PDs D unclear R n=1, C G PDs R n=1) | NR |
| **Saez-Landete et al.** | 6 | A Background theta/delta n=5  RPPs: RDA n=3 | NR |
| **Santos de Lima et al.** | 32 | A G slowing n=26, F slowing n=4, Normal n=6  PDs n= 8(G n=6, L n=2), ESz n=3, IIC n=8 | NR |
| **Skorin et al.** | 62 | A background slowing n= 62 (F slow waves n=13, G slow waves n=34, F C slowness n=3, theta background R n=17, G C slow waves delta n=62, G low voltage n=10), Normal n=9  ESz n=6 (NCSE n=2, IIC n=4)  RPPs n=3 | EEG at 3 weeks was associated with mortality at univariate (RR 4.2, 95%CI 1.4-13 p=0.013) and multivariate (RR 11, 95%CI 1.3-93, p=.0.027). |
| **Waters et al.** | 79 | G slowing n=78, F slowing n=11  ESz n=11 (IIC n=6, PDs/RDA n=5, GPD with triphasic morphology n=1), NCSE n=3 | NR |

Table Legend: ICU, intensive care unit; HIV, human immunodeficiency virus; EEG, electroencephalogram; cEEG, continuous electroencephalogram; CNS, central nervous system; AIS, acute ischemic stroke; ICH, intracranial hemorrhage; PRES, reversible posterior encephalopathy syndrome; NR, not reported; A, abnormal; PDs, periodic discharges; PDR, posterior dominant rhythm; PEDs, periodic epileptogenic discharge; RPPs, rhythmic and periodic patterns; RDA, rhythmic delta activity; SW, spike and wave/sharp and wave; G, generalized; F, focal; BI, bilateral independent; UI, unilateral independent; Mf, multifocal; ESz, electrographic seizures; ESE, electrographic status epilepticus; ECSE, electroclinical status epilepticus; ECSz, electroclinical seizures; NCSE, non-convulsive status epilepticus; C, continuous; D, discontinuous; BS, burst suppression; S, symmetric; AS, asymmetric; UR, un-reactive; R, reactive; IIC, ictal interictal continuum pattern, HR, hazard ratio; RR, relative risk, CI, confidence interval; sens, sensibility; spec, specificity, AUC, area under curve.

**Supplementary Item 2: Quality assessment**

Quality assessment was based on modified Newcastle-Ottawa Score by Murad et al. Quality score: Low = 0-2, Medium = 3-5, High = 6-8.

**NEWCASTLE - OTTAWA QUALITY ASSESSMENT SCALE CASE CONTROL STUDIES**

**Selection**

1. Is the case definition adequate?

a) yes, with independent validation

b) yes, eg record linkage or based on self reports c) no description

2) Representativeness of the cases

a) consecutive or obviously representative series of cases

b) potential for selection biases or not stated

3) Selection of Controls

a) community control

b) hospital controls

c) no description

4) Definition of Controls

a) no history of disease (endpoint)

b) no description of source

**Comparability**

1. Comparability of cases and controls on the basis of the design or analysis

a) study controls for _______________ (Select the most important factor)

b) study controls for any additional factor (This criteria could be modified to indicate specific

control for a second important factor)

**Exposure**

1. Ascertainment of exposure
2. secure record (eg surgical records)

b) structured interview where blind to case/control status

c) interview not blinded to case/control status

d) written self report or medical record only

e) no description

2) Same method of ascertainment for cases and controls

a ) yes

b) no

3) Non-Response rate

a) same rate for both groups

b) non respondents described

c) rate different and no designation

**NEWCASTLE - OTTAWA QUALITY ASSESSMENT SCALE COHORT STUDIES**

**Selection**

1) Representativeness of the exposed cohort

a) truly representative of the average neuromonitoring in COVID-19 (describe) in the community

b) somewhat representative of the average neuromonitoring in COVID-19 in the community
c) selected group of users eg nurses, volunteers

d) no description of the derivation of the cohort

2) Selection of the non exposed cohort

a) drawn from the same community as the exposed cohort

b) drawn from a different source

c) no description of the derivation of the non exposed cohort

3) Ascertainment of exposure

a) secure record (eg surgical records)

b) structured interview

c) written self report

d) no description

4) Demonstration that outcome of interest was not present at start of study

a ) yes

b) no

**Comparability**

1. Comparability of cohorts on the basis of the design or analysis
2. study controls for non-COVID-19 (select the most important factor)

b) study controls for any additional factor (This criteria could be modified to indicate specific

control for a second important factor.)

**Outcome**

1. Assessment of outcome

a) independent blind assessment

b) record linkage

c) self report

d) no description

2) Was follow-up long enough for outcomes to occur

a) yes (select an adequate follow up period for outcome of interest)

b) no

3) Adequacy of follow up of cohorts

a) complete follow up - all subjects accounted for

b) subjects lost to follow up unlikely to introduce bias - small number lost - > ____ % (select an adequate %) follow up, or description provided of those lost)

c) follow up rate < ____% (select an adequate %) and no description of those lost d) no statement.

**Supplementary Material - Table 4. Quality assessment**

| Author | Representative sample of relevant population | Study attrition (loss to follow-up and response rate) | Valid and reliable instrument for predictors | Objectively measured outcomes | Controlled for prior neurological comorbidities | Appropriate statistical analysis | Quality |
| --- | --- | --- | --- | --- | --- | --- | --- |
| Ayub et al. | 0 | 2 | 0 | 1 | 1 | 1 | Medium |
| Bellaia et al. | 0 | 1 | 1 | 1 | 1 | 1 | Medium |
| Besnard et al. | 0 | 0 | 0 | 0 | 0 | 0 | Low |
| Cecchetti et al. | 1 | 0 | 0 | 0 | 1 | 1 | Medium |
| Corazza et al. | 2 | 2 | 0 | 1 | 1 | 1 | High |
| Galantopou et al. | 1 | 0 | 0 | 1 | 1 | 1 | Medium |
| Karahan et al. | 2 | 1 | 1 | 1 | 0 | 1 | High |
| Lambreq et al. | 0 | 2 | 1 | 1 | 0 | 1 | Medium |
| Lin et al. | 0 | 2 | 0 | 1 | 1 | 1 | Medium |
| Louis et al. | 0 | 2 | 0 | 1 | 1 | 1 | Medium |
| Marcic et al. | 2 | 2 | 1 | 1 | 0 | 1 | High |
| Pasini et al. | 1 | 0 | 0 | 1 | 1 | 0 | Medium |
| Pastor et al. | 1 | 0 | 0 | 1 | 1 | 1 | Medium |
| Pati et al. | 1 | 1 | 0 | 0 | 0 | 0 | Low |
| Pellinen et al. | 0 | 2 | 1 | 1 | 1 | 1 | High |
| Petrescu et al. | 0 | 2 | 1 | 1 | 1 | 1 | High |
| Saez-Landete et al. | 0 | 1 | 0 | 0 | 1 | 1 | Medium |
| Santos de Lima et al. | 0 | 1 | 0 | 1 | 1 | 1 | Medium |
| Skorin et al. | 0 | 2 | 1 | 1 | 1 | 1 | High |
| Sonkaya et al. | 1 | 1 | 1 | 1 | 0 | 1 | Medium |
| Waters et al. | 0 | 2 | 1 | 1 | 1 | 1 | High |

**References**

1. Ayub N, Cohen J, Jing J, Jain A, Tesh R, Mukerji SS, Zafar SF, Westover MB, Kimchi EY. Clinical Electroencephalography Findings and Considerations in Hospitalized Patients With Coronavirus SARS-CoV-2. *The Neurohospitalist* (2021) **11**:204–213. doi:10.1177/1941874420972237

2. Bellavia S, Scala I, Luigetti M, Brunetti V, Gabrielli M, Zileri Dal Verme L, Servidei S, Calabresi P, Frisullo G, Della Marca G. Instrumental Evaluation of COVID-19 Related Dysautonomia in Non-Critically-Ill Patients: An Observational, Cross-Sectional Study. *J Clin Med* (2021) **10**:5861. doi:10.3390/jcm10245861

3. Besnard S, Nardin C, Lyon E, Debroucker T, Arjmand R, Moretti R, Pochat H. Electroencephalographic Abnormalites in SARS-CoV-2 Patients. *Front Neurol* (2020) **11**: doi:10.3389/fneur.2020.582794

4. Cecchetti G, Vabanesi M, Chieffo R, Fanelli G, Minicucci F, Agosta F, Tresoldi M, Zangrillo A, Filippi M. Cerebral involvement in COVID-19 is associated with metabolic and coagulation derangements: an EEG study. *J Neurol* (2020) **267**:3130–3134. doi:10.1007/s00415-020-09958-2

5. Corazza LA, Tatsh JFS, Barros MP, Queiroz AP de, Batista LLR, Aidar MB, Baldocchi MA, Rocha MSG, Brucki SMD. Electroencephalographic findings among inpatients with COVID-19 in a tertiary hospital from a middle-income country. *Arq Neuropsiquiatr* (2021) **79**:315–320. doi:10.1590/0004-282x-anp-2020-0555

6. Galanopoulou AS, Ferastraoaru V, Correa DJ, Cherian K, Duberstein S, Gursky J, Hanumanthu R, Hung C, Molinero I, Khodakivska O, et al. EEG findings in acutely ill patients investigated for SARS‐CoV‐2/COVID‐19: A small case series preliminary report. *Epilepsia Open* (2020) **5**:314–324. doi:10.1002/epi4.12399

7. Karahan M, Demirtaş AA, Hazar L, Erdem S, Ava S, Dursun ME, Keklikçi U. Autonomic dysfunction detection by an automatic pupillometer as a non-invasive test in patients recovered from COVID-19. *Graefe’s Arch Clin Exp Ophthalmol* (2021) doi:10.1007/s00417-021-05209-w

8. Lambrecq V, Hanin A, Munoz-Musat E, Chougar L, Gassama S, Delorme C, Cousyn L, Borden A, Damiano M, Frazzini V, et al. Association of Clinical, Biological, and Brain Magnetic Resonance Imaging Findings With Electroencephalographic Findings for Patients With COVID-19. *JAMA Netw Open* (2021) **4**:e211489. doi:10.1001/jamanetworkopen.2021.1489

9. Lin L, Al‐Faraj A, Ayub N, Bravo P, Das S, Ferlini L, Karakis I, Lee JW, Mukerji SS, Newey CR, et al. Electroencephalographic Abnormalities are Common in <scp>COVID</scp> ‐19 and are Associated with Outcomes. *Ann Neurol* (2021) **89**:872–883. doi:10.1002/ana.26060

10. Louis S, Dhawan A, Newey C, Nair D, Jehi L, Hantus S, Punia V. Continuous electroencephalography characteristics and acute symptomatic seizures in COVID-19 patients. *Clin Neurophysiol* (2020) **131**:2651–2656. doi:10.1016/j.clinph.2020.08.003

11. Marcic M, Marcic L, Marcic B, Capkun V, Vukojevic K. Cerebral Vasoreactivity Evaluated by Transcranial Color Doppler and Breath-Holding Test in Patients after SARS-CoV-2 Infection. *J Pers Med* (2021) **11**:379. doi:10.3390/jpm11050379

12. Pasini E, Bisulli F, Volpi L, Minardi I, Tappatà M, Muccioli L, Pensato U, Riguzzi P, Tinuper P, Michelucci R. EEG findings in COVID-19 related encephalopathy. *Clin Neurophysiol* (2020) **131**:2265–2267. doi:10.1016/j.clinph.2020.07.003

13. Pastor J, Vega-Zelaya L, Martín Abad E. Specific EEG Encephalopathy Pattern in SARS-CoV-2 Patients. *J Clin Med* (2020) **9**:1545. doi:10.3390/jcm9051545

14. Pati S, Toth E, Chaitanya G. Quantitative EEG markers to prognosticate critically ill patients with COVID-19: A retrospective cohort study. *Clin Neurophysiol* (2020) **131**:1824–1826. doi:10.1016/j.clinph.2020.06.001

15. Pellinen J, Carroll E, Friedman D, Boffa M, Dugan P, Friedman DE, Gazzola D, Jongeling A, Rodriguez AJ, Holmes M. Continuous EEG findings in patients with COVID‐19 infection admitted to a New York academic hospital system. *Epilepsia* (2020) **61**:2097–2105. doi:10.1111/epi.16667

16. Petrescu A-M, Taussig D, Bouilleret V. Electroencephalogram (EEG) in COVID-19: A systematic retrospective study. *Neurophysiol Clin* (2020) **50**:155–165. doi:10.1016/j.neucli.2020.06.001

17. Sáez-Landete I, Gómez-Domínguez A, Estrella-León B, Díaz-Cid A, Fedirchyk O, Escribano-Muñoz M, Pedrera-Mazarro A, Martín-Palomeque G, Garcia-Ribas G, Rodríguez-Jorge F, et al. Retrospective Analysis of EEG in Patients With COVID-19: EEG Recording in Acute and Follow-up Phases. *Clin EEG Neurosci* (2021)155005942110359. doi:10.1177/15500594211035923

18. Santos de Lima F, Issa N, Seibert K, Davis J, Wlodarski R, Klein S, El Ammar F, Wu S, Rose S, Warnke P, et al. Epileptiform activity and seizures in patients with COVID-19. *J Neurol Neurosurg Psychiatry* (2021) **92**:565–566. doi:10.1136/jnnp-2020-324337

19. Skorin I, Carrillo R, Perez CP, Sanchez N, Parra J, Troncoso P, Uribe-San-Martin R. EEG findings and clinical prognostic factors associated with mortality in a prospective cohort of inpatients with COVID-19. *Seizure* (2020) **83**:1–4. doi:10.1016/j.seizure.2020.10.007

20. Sonkaya AR, Özturk B, Karadas Ö. Cerebral hemodynamic alterations in patients with Covid-19. *Turkish J Med Sci* (2021) **51**:435–439. doi:10.3906/sag-2006-203

21. Waters BL, Michalak AJ, Brigham D, Thakur KT, Boehme A, Claassen J, Bell M. Incidence of Electrographic Seizures in Patients With COVID-19. *Front Neurol* (2021) **12**: doi:10.3389/fneur.2021.614719
